# Metagenomic and Microscopic Analysis of Magnetotactic Bacteria in Tangyin Hydrothermal Field of Okinawa Trough

**DOI:** 10.3389/fmicb.2022.887136

**Published:** 2022-06-10

**Authors:** Si Chen, Min Yu, Wenyan Zhang, Kuang He, Hongmiao Pan, Kaixuan Cui, Yicong Zhao, Xiao-Hua Zhang, Tian Xiao, Wuchang Zhang, Long-Fei Wu

**Affiliations:** ^1^CAS Key Laboratory of Marine Ecology and Environmental Sciences, Institute of Oceanology, Chinese Academy of Sciences, Qingdao, China; ^2^Laboratory for Marine Ecology and Environmental Science, Qingdao National Laboratory for Marine Science and Technology, Qingdao, China; ^3^University of Chinese Academy of Sciences, Beijing, China; ^4^Center for Ocean Mega-Science, Chinese Academy of Sciences, Qingdao, China; ^5^College of Marine Life Sciences, Ocean University of China, Qingdao, China; ^6^International Associated Laboratory of Evolution and Development of Magnetotactic Multicellular Organisms (LIA-MagMC), CNRS-CAS, Qingdao, China; ^7^Key Lab of Submarine Geosciences and Prospecting Techniques, Frontiers Science Center for Deep Ocean Multispheres and Earth System, MOE and College of Marine Geosciences, Ocean University of China, Qingdao, China; ^8^Aix Marseille University, CNRS, LCB, Marseille, France

**Keywords:** magnetotactic bacteria, 16S rRNA gene, magnetosome genes, magnetofossil, hydrothermal field

## Abstract

Magnetotactic bacteria (MTB) have been found in a wide variety of marine habitats, ranging from intertidal sediments to deep-sea seamounts. Deep-sea hydrothermal fields are rich in metal sulfides, which are suitable areas for the growth of MTB. However, MTB in hydrothermal fields have never been reported. Here, the presence of MTB in sediments from the Tangyin hydrothermal field was analyzed by 16S rRNA gene amplicon analysis, metagenomics, and transmission electron microscopy. Sequencing 16S rRNA gene yielded a total of 709 MTB sequences belonging to 20 OTUs, affiliated with *Desulfobacterota, Alphaproteobacteria*, and *Nitrospirae*. Three shapes of magnetofossil were identified by transmission electron microscopy: elongated-prismatic, bullet-shaped, and cuboctahedron. All of these structures were composed of Fe_3_O_4_. A total of 121 sequences were found to be homologous to the published MTB magnetosome-function-related genes, and relevant domains were identified. Further analysis revealed that diverse MTB are present in the Tangyin hydrothermal field, and that multicellular magnetotactic prokaryote (MMPs) might be the dominant MTB.

## Introduction

Magnetotactic bacteria (MTB) represent a group of prokaryotes that can migrate along geomagnetic field lines (Blakemore, 1975; Bellini, 2009). MTB are diverse in their morphology, phylogeny, and physiology. There are single-cell forms (cocci, ovoid, rod, curved rod, and spirillum) and multicellular forms that are known as the multicellular magnetotactic prokaryotes (MMPs). The MMPs can be divided into two types based on their shape: spherical MMPs (sMMPs) and ellipsoidal MMPs (eMMPs) (Bazylinski et al., 2013; Amor et al., 2020). MTB exhibits great taxonomic diversity. Most are phylogenetically affiliated with phyla of *Proteobacteria, Desulfobacterota* (previously known as *Deltaproteobacteria*), *Nitrospirae, Planctomycetes*, the candidate phylum *Omnitrophica*, and the candidate phylum *Latescibacteria* (Bazylinski et al., 2013; Lin et al., 2018). Recent studies of reconstructed metagenome-assembled MTB genomes have expanded our knowledge of the taxonomy of MTB, several of which belonged to the phyla *Nitrospinota*, UBA10199, *Bdellovibrionota, Bdellovibrionata_B, Fibrobacterota, Riflebacteria* phyla, *Elusimicrobia*, and *Candidatus* Hydrogenedentes (Lin et al., 2020; Uzun et al., 2020).

The MTB can use intracellular biomineralization to synthesize special organelles called magnetosomes, which help them find and remain near suitably chemically stratified water columns or sediments. Magnetosomes are composed of magnetite or/and greigite (Zhang et al., 2014) and exhibit different shapes (e.g., elongated-prismatic, bullet/tooth-shaped, and cuboctahedron) (Bazylinski et al., 2013). There is a strong correlation between the morphology of biogenetic magnetosomes and MTB phylogeny (Li et al., 2020b). *Alphaproteobacteria* MTB always synthesizes cuboctahedral or elongated-prismatic magnetite magnetosomes, while MTB with bullet-shaped magnetite crystals is associated with the *Desulfobacterota, Nitrospirae*, and the candidate phylum *Omnitrophica* (Lin et al., 2014; Amor et al., 2020). *Desulfobacterota* MTB can also biomineralize greigite crystals of diverse morphologies (Lefèvre et al., 2011; Zhang et al., 2014). Inside the cell, magnetosomes are arranged as single chains, double chains, or multiple chains, and disorderly arrangements have also been reported (Amor et al., 2020). Magnetosome formation in MTB is a biomineralization process that is strictly controlled by conserved genes found in magnetosome gene clusters (MGCs) (Lin et al., 2020). Generally, there are four steps in the formation of magnetosomes, which arrange in chains: cytoplasmic membrane invagination forms vesicles, proteins are targeted to the vesicle (magnetosome) membrane, iron is transported to vesicles (membranous invagination of magnetosomes) and mineralized into magnetite crystals, and the crystals are assembled into chains of magnetosomes (Uebe and Schüler, 2016). Studies on pure cultures of the MTB, like *Magnetospirillum gryphiswaldense* MSR-1, revealed that the MGC genes are found in five operons: the *mms6* operon, the *mamGFDC* operon, the *mamAB* operon, the *mamXY* operon, and the *feoAB1* operon (Uebe and Schüler, 2016). Magnetosome genes that compose MGCs of different MTB species are not identical, as reflected in species-level differences in magnetosome type. However, almost all MGCs contain the *mamAB* operon (Lefèvre and Wu, 2013). This operon contains the core gene, *mamABEKMOPQI*, which is thought to play an important role in magnetosome formation (Lefèvre and Wu, 2013; Lin et al., 2018). After the death of a magnetotactic bacterium, the magnetosomes are released; over time, they accumulate in sediments, forming fossil magnetosomes (magnetofossils) (Lin et al., 2014). The magnetic mineral ultrastructure, morphology, composition, size, and other characteristics of magnetofossils can be observed and effectively distinguished using a transmission electron microscope (TEM) (Li et al., 2020a). The study of magnetofossils can provide paleoecological and paleoenvironmental information (Hesse, 1994).

The MTB is widespread in sediments and water at the oxic-anoxic interface (OAI, previously called the OATZ) of freshwater, brackish, marine, and hypersaline environments (Bazylinski et al., 2013). The abundance of marine MTB is usually higher in the intertidal zone, where most reports indicate that magnetotactic cocci are relatively common and the dominant species of MTB (Lin et al., 2012; Abreu et al., 2016). Most of these studied environments have near-neutral pH, moderate temperatures, and are concentrated in the Northern hemisphere. However, there are some reports of MTB in special habitats, including mangrove swamps, coral reefs, seamounts, deep seas, etc. (Torres de Araujo et al., 1986; Dong et al., 2016; Liu et al., 2017; Teng et al., 2018). Vibrio and rod-shaped MTB, but not cocci, have been found in hemipelagic sediments of the Santa Barbara Basin (Stolz et al., 1986). Other studies indicated that the MTB found in extreme environments often belonged to *Desulfobacterota* and *Nitrospirae* (Abreu et al., 2007; Nash, 2008; Lefèvre et al., 2010). In addition, biogenic magnetite, with elongated octahedral and prismatic morphologies in ferromanganese nodules, has also been reported (Hassan et al., 2020).

In deep-sea hydrothermal fields, which are rich in metal sulfides, microorganisms mainly obtain energy from reductive sulfur oxidation (McCollom and Shock, 1997; Amend et al., 2011; Meier et al., 2019), and chemolithoautotrophic microorganisms represent the only primary producers (Jannasch and Mottl, 1985). Simultaneously, most cultured MTB can grow chemolithoautotrophically using reductive sulfides. *Desulfobacterota* MTB are sulfate-reducing anaerobes that grow only chemoorganoheterotrophically (Bazylinski et al., 2013). MTB might dwell in and adapt to hydrothermal environments. To assess this possibility, we combined 16S rRNA gene amplicon and metagenomic data and TEM observations to study the presence, diversity, and characteristics of MTB in the Tangyin hydrothermal field of Okinawa Trough.

## Materials and Methods

### Sample Collection

The Tangyin hydrothermal field is located atop an upland found 38 km northeast of Yonaguni Knoll IV field, at the southern end of the Okinawa Trough. Surface sediment samples were collected by a box sampler during a cruise conducted by the R/V Kexue in May 2014. All sediment samples were quick-frozen in liquid nitrogen and stored at −80°C until laboratory analysis.

### 16S rRNA Gene Sequencing and Analysis

Total genomic DNA was extracted from sediment samples as described by Zhou et al. (1996). The V3–V4 regions of the 16S rRNA gene were amplified using universal primer sets 338F and 806R. PCR products were purified, quantified, and paired-end sequencing was performed on the Illumina MiSeq PE300 platform at Majorbio Bio-Pharm Technology Co., Ltd. (Shanghai, China). Detailed protocols were previously published by Wang et al. (2018). The paired-end reads of fastq files were merged and quality-filtered using Usearch (version 8.1) (Edgar, 2016). The filtered reads were clustered into operational taxonomic units (OTUs), using UPARSE with the threshold set to 97% (Edgar, 2013). Representative reads for OTUs were aligned with the GenBank nucleic acid database (NT) using BLASTn, and MTB-related OTUs were screened using an identity threshold of 90% (Altschul et al., 1990). The CLUSTALW multiple alignment software was used for sequence alignment (Larkin et al., 2007). The phylogenetic tree was constructed based on the neighbor-joining method using MEGA6 (Tamura et al., 2013) with the bootstrap *p* value of 1,000.

### Rock Magnetic Measurements

We measured the isothermal remanent magnetization (IRM) acquisition curve and first-order reversal curves (FORC) on the bulk sample using a vibrating sample magnetometer (VSM, Princeton Measurements Corporation's MicroMag™ 3900) at the Institute of Geology and Geophysics, Chinese Academy of Sciences (IGGCAS) (Roberts et al., 2000; Kruiver et al., 2001; Egli et al., 2010). For the IRM acquisition curve, the field was added from 10 μT to 1 T with 120 data points in a log distribution of the field steps and the averaging time is 1 s for each data point. Coercivity unmixing analyses were conducted using the Max Unmix model (Maxbauer et al., 2016). For the FORC diagram, the saturation field is set to 1 T and 200 partial hysteresis curves were measured with 350 ms averaging time. We used FORCinel software (v 3.06) to create a FORC diagram (Harrison and Feinberg, 2008). The FORC were smoothed using VARIFORC parameters: *S*_c0_ = 6, *S*_b0_ = 5, *S*_c1_ = *S*_b1_ = 8.

### Magnetofossils Observation

The so-called magnetic fingers were used to extract magnetofossils, as previously described (Von Dobeneck et al., 1987; He and Pan, 2020). We made a minor modification based on the method mentioned above. Briefly, 2 ml of sediment and 0.1 g sodium hexametaphosphate were mixed with 30 ml Milli-Q water in a 50-ml centrifuge tube, and a magnetic finger was put into the mixture for at least 12 h. Magnetic minerals were washed from the surface of the magnetic finger into a 15-ml centrifuge tube using Milli-Q water. Two circular magnets were attached to the outside of the 15-ml centrifuge tube, and the enriched particles were shaken until they were evenly distributed throughout the tube and then allowed to settle for at least 12 h. A Pasteur tube was used to recover the magnetic particles that were adsorbed on the inner wall and transfer them to a 1.5-ml centrifuge tube. After rinsing the tube three times using Milli-Q water, 30 μl of anhydrous ethanol was used to re-suspend the magnetic particles. Then, 8 μl of this suspension was dropped onto a copper double grid for TEM observation.

The morphological characteristics of the magnetofossils were observed using a Hitachi H8100 microscope operating at 100 kV at the Institute of Oceanology, Chinese Academy of Sciences (IOCAS). High-resolution transmission electron microscopy (HRTEM), selected area electron diffraction (SAED), and X-ray energy-dispersive spectroscopy (XEDS) were obtained using a JEOL JEM-2100 TEM operated at 200 kV at the Institute of Geology and Geophysics, Chinese Academy of Sciences (IGGCAS).

### Metagenome Sequencing and MGCs Analysis

The genomic DNA was sequenced in BGI Co., Ltd. (Wuhan, China) *via* shotgun sequencing on the Illumina HiSeq platform (150 bp paired-end strategy). The raw sequencing data were filtered and trimmed to generate high-quality clean data. Clean reads were assembled using the IDBA-UD software (Peng et al., 2012). Genes were predicted using Prodigal (Hyatt et al., 2010) and aligned with known MGC genes using BLASTp, with thresholds of 35% for similarity and 50% for gene coverage. MGC genes (*mam, mad, feo, mms, man*) of MTB strains were downloaded from the MAGE website (Vallenet et al., 2019). All genes were manually checked, and putative magnetosome genes with higher homology to known MGC genes were selected for further analysis. The abundances of these putative magnetosome genes were calculated, and the Pfam database was used to predict the domains of the selected genes and known magnetosome genes (Mistry et al., 2021). At the same time, some selected putative magnetosome gene sequences have higher similarities with known homologous genes. These sequences were used as representative sequences to construct phylogenetic trees. The construction method is the same as above. The sequences homologous to the two known MTB magnetome genes with the most abundant number and species were selected, and the sequences with the highest similarity to the known sequences in each putative magnetome gene were selected for gene organization comparison. Jalview was used to analyze the conserved regions of predictive protein sequences of magnetosome genes (Waterhouse et al., 2009).

## Results

### MTB Community Analysis

**Figure 1:**
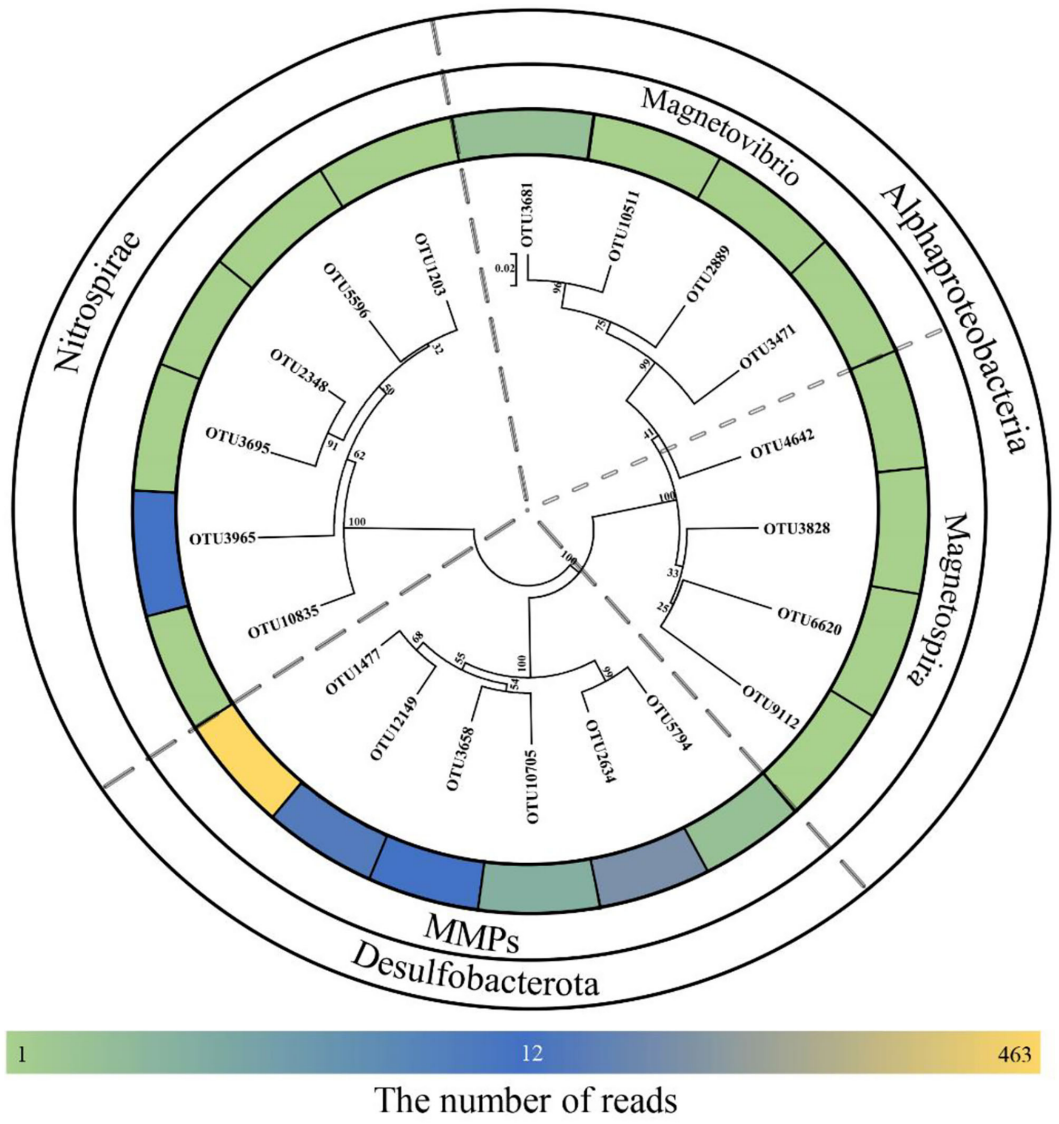
Neighbor-joining tree for environmental magnetotactic bacteria based on 16S rRNA gene sequences. Scale bar: 0.02.

Using high-throughput sequencing, we obtained a total of 153,056 tags from the Tangyin hydrothermal field sediment samples, corresponding to a total of 3,115 OTUs using the threshold of 97% sequence identity. After alignment to published MTB 16S rRNA gene sequences, a total of 709 sequences were screened out (0.46% of all tags). These sequences belonged to 20 OTUs (0.64% of all OTUs) (Supplementary Table S1). Of them, eight (10 reads) belonged to genera *Magnetovibrio* and *Magnetospira* in the class of *Alphaproteobacteria*, six (17 reads) belonged to *Nitrospirae* MTB, and six (682 reads) belonged to MMPs affiliated with *Desulfobacterota*. Notably, two dominant MMP OTUs accounted for 463 reads and 146 reads and were highly similar to sMMP *Ca*. Magnetomorum litorale. Of the remaining four MMP-related OTUs, three (60 reads) were most similar to sMMP *Ca*. Magnetoglobus multicellularis Araruama and one (13 reads) was similar to eMMP *Ca*. Magnetananas rongchenensis (Table 1). Results of the phylogenetic tree based on representative sequences of the OTUs were consistent with our sequence alignment results (Figure 1).

**Table 1:** Numbers of OTUs and reads corresponding to different species of magnetotactic bacteria.

			**OTUs**	**Reads**
*Desulfobacterota*	sMMPs	*Ca*. Magnetomorum litorale	2	609
		*Ca*. Magnetoglobus multicellularis Araruama	3	60
	eMMPs	*Ca*. Magnetananas rongchenensis	1	13
*Nitrospirae*		*Magnetobacterium* sp. P1B_23	2	13
		*Ca. Magnetobacterium bavaricum*	3	3
		Uncultured magnetotactic rod MHB-1	1	1
*Alphaproteobacteria*	Magnetovibrio	*Magnetovibrio blakemorei* MV-1	4	6
	Magnetospira	*Magnetospira* sp. QH-2	3	3
		*Magnetospira thiophila* MMS-1	1	1
Total			20	709
Bacteria			3,115	153,056
MTB percentage of bacteria			0.64%	0.46%

**Figure 2:**
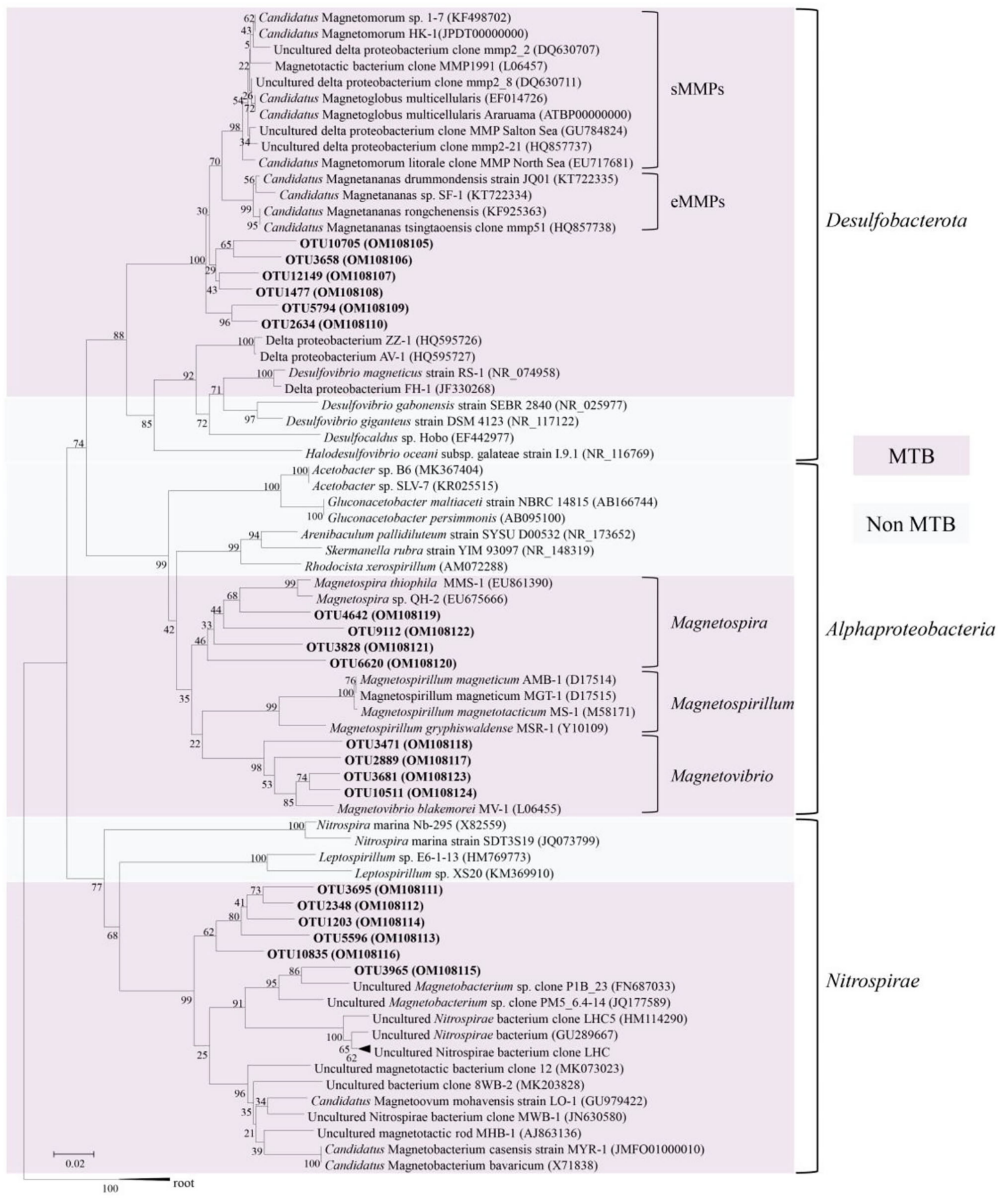
Phylogenetic tree constructed based on 16S rRNA sequence gene reads related to magnetotactic bacteria. The sequences determined in this study are shown in bold text. GenBank accession numbers of the sequences used are indicated in parentheses. Scale bar: 0.02.

The 16S rRNA gene sequences clustered six OTUs into a branch within *Desulfobacterota*, which was branched with known MMPs but formed a different group (Figure 2). The five MTB-related OTUs belonging to the phylum *Nitrospirae* were also found in a different clade from the known MTB in the phylogenetic tree, while OTU3965 was clustered in the same clade with the known thermophilic MTB (Figure 2). Besides, the MTB-related OTUs affiliated with *Magnetovibrio* and *Magnetospira* were also found in the same branch as the known MTB (Figure 2).

### Detection of Magnetofossils

**Figure 3:**
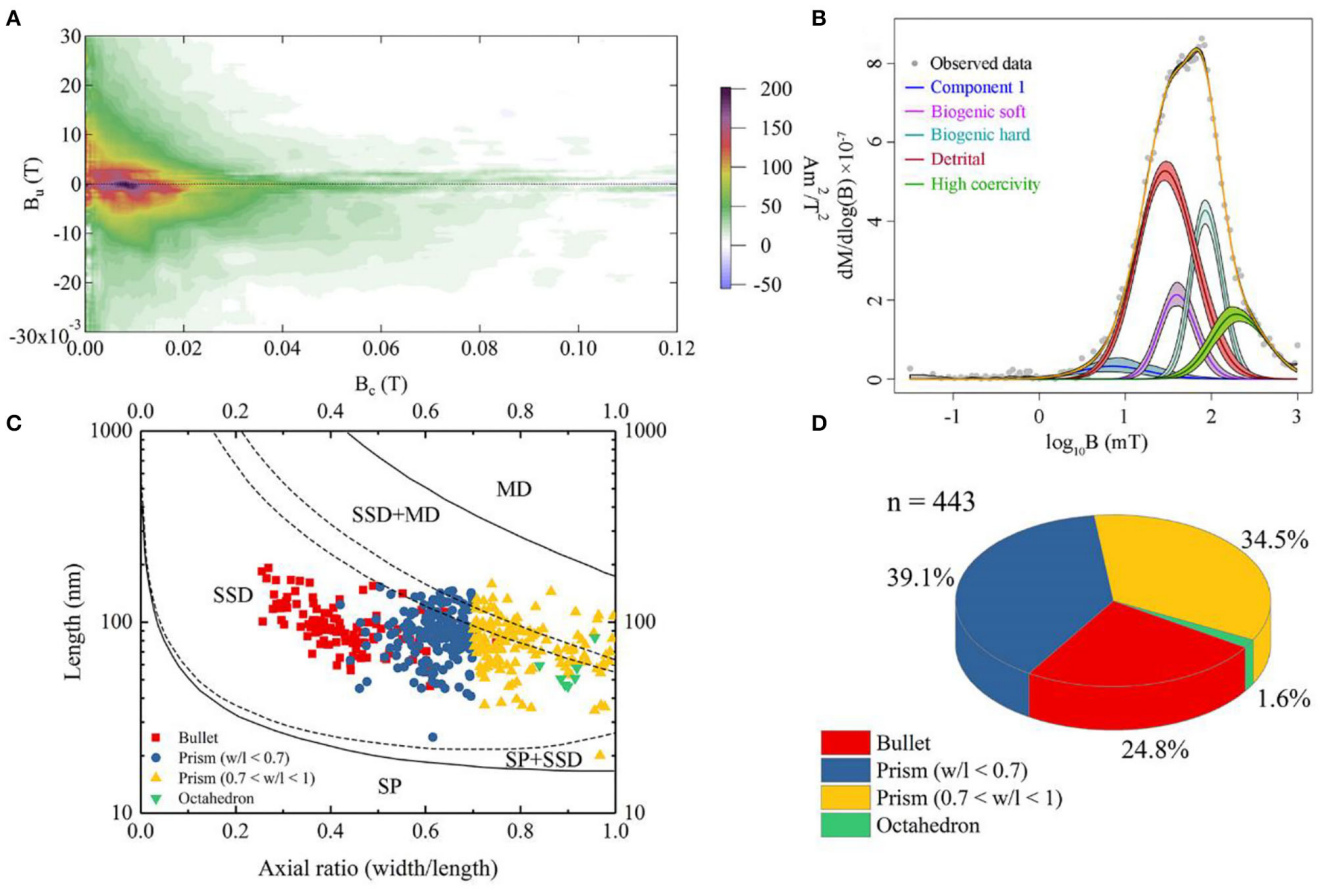
Rock magnetic and statistical data on magnetofossil morphologies for the sediment. **(A)** First-order reversal curve (FORC) diagram. The *B*_c_ and *B*_u_ axis indicates coercivity and magnetostatic interaction, respectively. **(B)** Isothermal remanent magnetization (IRM)-unmixing result. The horizontal axis stands for coercivity (expressed on a base 10 logarithmic scale), and the gradient of the IRM acquisition curve (yellow) is fitted by lognormal distributions. The different color stands for different components; Purple and turquoise curves are biogenic soft and hard components, respectively. Red and green curves are detrital and high coercivity components, respectively. The blue curve may indicate coarse-grained minerals. **(C)** Magnetofossil size distributions. Red square, navy blue dot, and yellow triangle represent bullet-shaped, elongated prism, and short prism, respectively. The green triangle denotes octahedral magnetofossil. The domain-state phase diagram is modified after Muxworthy and Williams (2009). SP, SD, SSD, and MD represent superparamagnetic, single domain, stable single domain, and multidomain, respectively. **(D)** Statistics based on panel **(C)**. The colors representing different morphotypes of magnetofossils are consistent with those in panel **(C)**.

The FORC diagram does not show a clear central ridge which indicates non-interacting uniaxial single domain grains (Figure 3A). It suggests that the bulk sample probably contains multi-domain (MD) and/or vortex magnetic minerals and magnetofossils with broken chains. We decomposed the sample into five components using IRM unmixing analysis, including component 1, biogenic soft (BS, with a median coercivity of 41.1 ± 1 mT), biogenic hard (BH, with a median coercivity of 88.4 ± 1 mT), detrital (with a median coercivity of 30.1 ± 1 mT), and high coercivity component (with a median coercivity of 217.3 ± 1 mT). The dispersion parameter (DP) of BS and BH components is 0.21 and 0.18, respectively (Figure 3B). Component 1 might indicate coarse magnetic minerals with lower coercivity (7.4 ± 1.1 mT). The high coercivity components probably indicate that the sample contains hematite or goethite. Detrital magnetic minerals are the dominant component which contribute 48.8% to the remanent magnetization of the bulk sample (Figure 3B). The proportions of BS and BH, which usually represents isotropic and elongated magnetofossils, are 12.4% and 21.1%, respectively.

**Figure 4:**
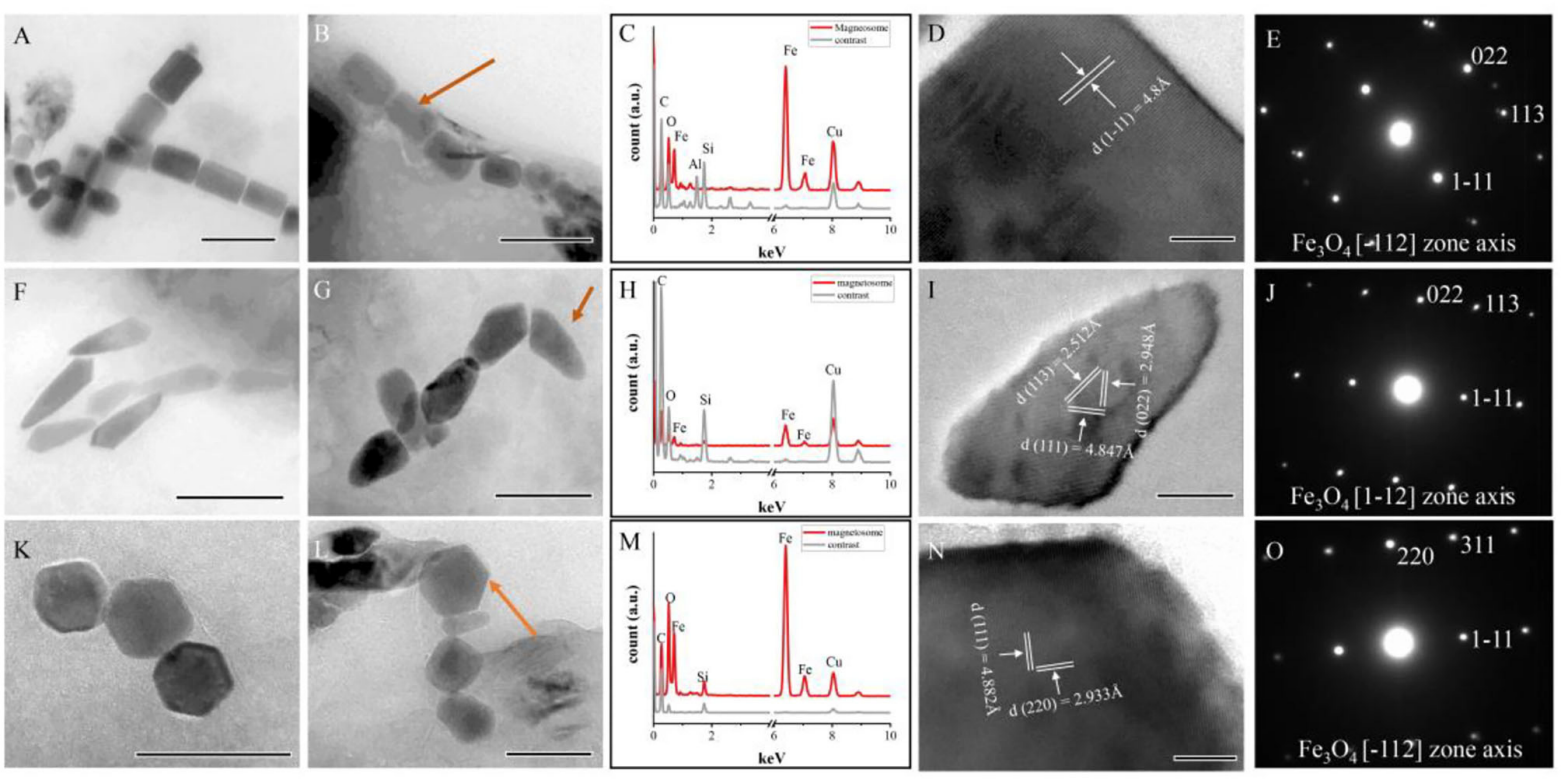
Characteristics of magnetofossils with three main shapes. **(A,B)** Elongated-prismatic magnetofossils. **(C–E)** Energy dispersive X-ray spectra **(C)**, HRTEM image **(D)**, and electron diffraction patterns **(E)** of the magnetofossil indicated by an arrow in panel **(B)**. **(F,G)** Bullet-shaped magnetofossils. **(H–J)** Energy dispersive X-ray spectra **(H)**, HRTEM image **(I)**, and electron diffraction patterns **(J)** of the magnetofossil indicated by an arrow in panel **(G)**. **(K,L)** Cuboctahedron magnetofossils. **(M–O)** Energy dispersive X-ray spectra **(M)**, HRTEM image **(N)**, and electron diffraction patterns **(O)** of the magnetofossil are indicated by an arrow in panel **(L)** (cuboctahedron magnetofossil). Scale bars **(A,B,F)** 200 nm; **(G,K,L)** 100 nm; **(I)** 20 nm; and **(D,N)** 10 nm.

Subsequently, a total of 443 magnetofossils were observed by TEM. Three different magnetofossil morphologies were recognized: elongated-prismatic, bullet-shaped, and cuboctahedral (Figures 4A,B,F,G,K,L). Most magnetofossils fall within a stable single domain (SSD) size range (Figure 3C). Prismatic magnetofossils (84 ± 25 × 59 ± 18 nm, *n* = 326) were the dominant type, accounting for 73.6% of the total magnetofossils (Figure 3D). Here, we define prisms with an axial ratio smaller than 0.7 to be elongated prismatic magnetofossils while those with an axial ratio larger than 0.7 to be short prisms. Bullet-shaped (98 ± 29 × 40 ± 10 nm, *n* = 110) magnetofossils accounted for 24.8% of the total, while the cuboctahedron type accounted for only 1.6% (56 ± 12 × 51 ± 12 nm, *n* = 7). Energy-dispersive X-ray analysis indicated that the variously shaped magnetofossils were all composed of iron and oxygen (Figures 4C,H,M). Measurement of the crystal lattice and analysis of the electron diffraction pattern showed that the particles were magnetite (Figures 4D,E,I,J,N,O).

### Analysis of Homologous Sequences of Magnetosome Genes

**Figure 5:**
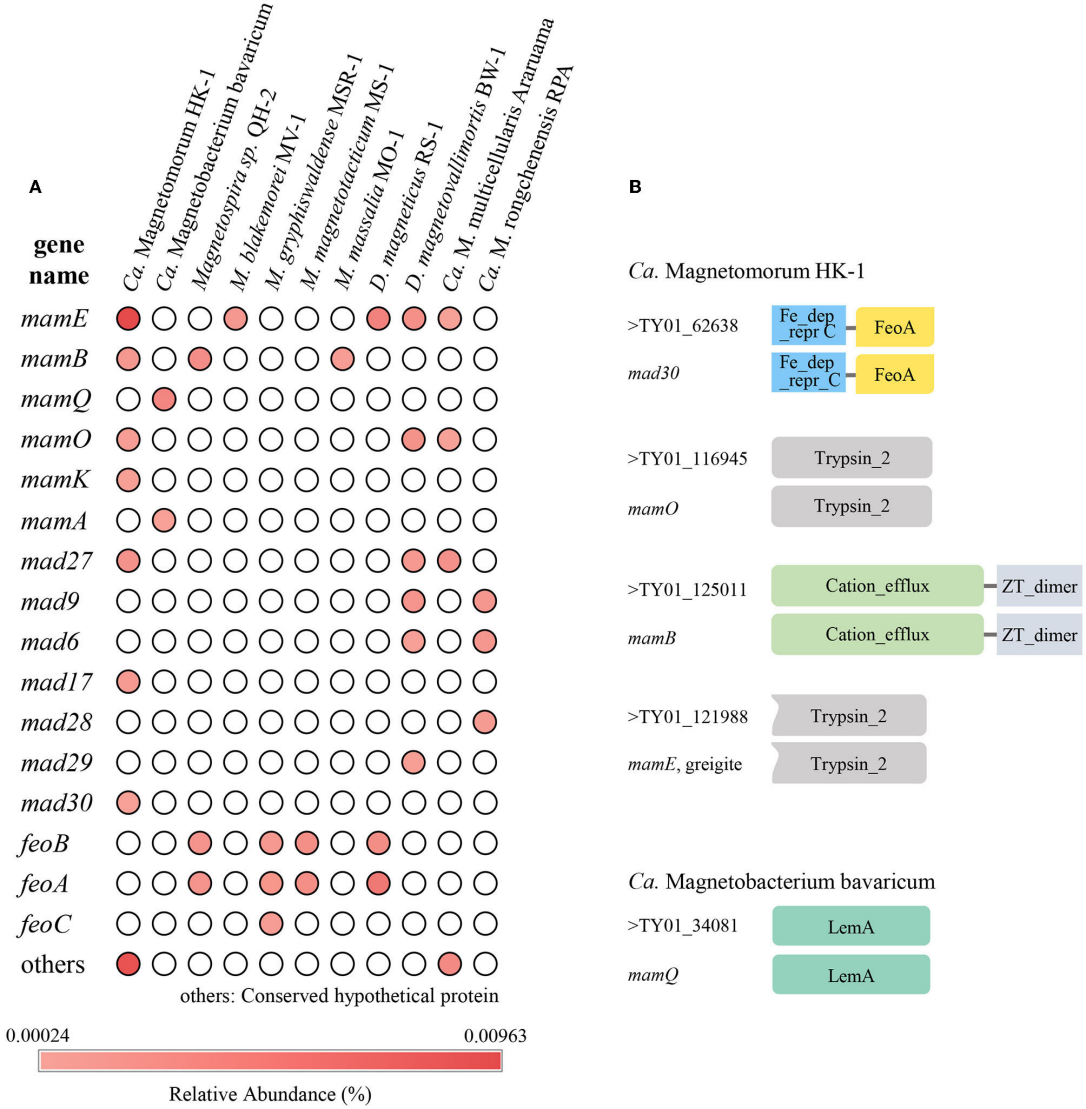
Homologous magnetosome genes analyses. **(A)** Relative abundance of homologous sequences of magnetosome genes in the sampled sediment. **(B)** Structural domain prediction for sequences that were homologous to *Ca*. Magnetomorum HK-1 and *Ca. Magnetobacterium bavaricum*. Fe_dep_repr C, Iron dependent repressor, metal binding, and dimerization domain; FeoA, FeoA domain; Trypsin_2, trypsin-like peptidase domain; Cation_efflux, Cation efflux family; ZT_dimer, Dimerization domain of Zinc Transporter; LemA, LemA protein family domain.

Through functional annotation and comparison with known MGC genes, a total of 121 homologous sequences of magnetosome genes were found (Supplementary Table S2), yielding a relative abundance of 0.078%. These homologous sequences of magnetosome genes were assigned to different genera or species based on similarity and showed relatively high similarity to 11 species of MTB. The homologous magnetosome genes with high similarity to MMPs (*Ca*. Magnetomorum HK-1, *Ca*. Magnetoglobus multicellular Araruama, *Ca*. Magnetananas Rongchenensis) accounted for 44.2% with 0.035% relative abundance. Also, the sequences that were homologous to *Ca*. Magnetomorum HK-1 had the highest relative abundance (0.025%). Four types of magnetosome genes were identified: *mam, mad, feo*, and others (i.e., conserved hypothetical protein and magnetosome-associated genes). The homologous sequence of *mam* gene clusters had the highest (0.037%) relative abundance among the four types; the homologous sequences of *mamE* accounted for the majority (0.024%), and the homologous sequences of *mamABOKQ* were also found. The various homologous sequences of *mad* genes (*mad6, mad9, mad17, mad28, mad29*, and *mad30*) were found, as were three *feo* genes (*feoA, feoB*, and *feoC*-*like*) (Figure 5A).

Domain prediction was carried out on the homologous protein sequences. Most of the homologous protein sequences had the same domains as known magnetosome proteins. For example, TY01_125011, which was homologous to *mamB* of *Ca*. Magnetomorum HK-1, the homologous MamB protein, was predicted to have ZT-dimer and cation efflux domains, while TY01_34081, which was homologous to *mamQ* of *Ca*. Magnetobacterium Bavaricum, the homologous MamQ protein had a LemA domain (Figure 5B).

## Discussion

Since MTB was first discovered independently by Bellini and Blakemore (Blakemore, 1975; Bellini, 2009), the phylogenetic information reported for them has been based primarily on 16S rRNA gene sequence analysis. As it has proven to be difficult to cultivate MTB, the 16S rRNA gene analysis has been used to infer the diversity and phylogenetic affiliations of MTB in various environments (Lin et al., 2018; Teng et al., 2018; Amor et al., 2020). Tan et al. (2021) analyzed MTB communities in tropical marine environments using the 16S rRNA genes from the metagenomic analysis. MTB at seamounts was also identified by 16S rRNA gene analysis (Liu et al., 2017). Dong et al. (2016) used the 16S rRNA gene and magnetofossil analyses to infer the existence of MTB in deep-sea surface sediments. Most mature biogenic magnetosomes are single-domain and arranged in chains in most MTB cells. According to these two basic characteristics, combined with the size, crystallographic structure, and composition of the magnetosomes, TEM can be used to identify magnetofossils effectively (Kopp and Kirschvink, 2008; Li et al., 2020a). Previous studies found that biogenic magnetite was distributed widely in deep-sea sediments (Petersen et al., 1986; Yamazaki et al., 2019; He and Pan, 2020; Usui and Yamazaki, 2021), and the proportions of bullet-shaped magnetofossils increased in relatively reductive and less oxic environments, while isotropic magnetofossils dominated in relatively oxic environments (Hesse, 1994; Yamazaki and Kawahata, 1998; Yamazaki and Shimono, 2013; He and Pan, 2020; Lu et al., 2021). Recently, the known extent of MTB diversity has undergone a significant expansion due to the identification of magnetosome gene cluster (MGC)-containing genomes and studies screening for sequences homologous to known MGC genes (Lin et al., 2020; Uzun et al., 2020). Lin et al. (2020) performed a large-scale reconstruction of metagenome-assembled MTB genomes from diverse ecosystems, and 13 bacterial phyla were detected, six of which were not previously known, including MTB. Thus, the existing literature indicates that analyses of 16S rRNA gene sequences, magnetofossil diversity, and magnetosome gene-homologous sequences obtained from an environment can be used to predict the existence of MTB diversity.

**Figure 6:**
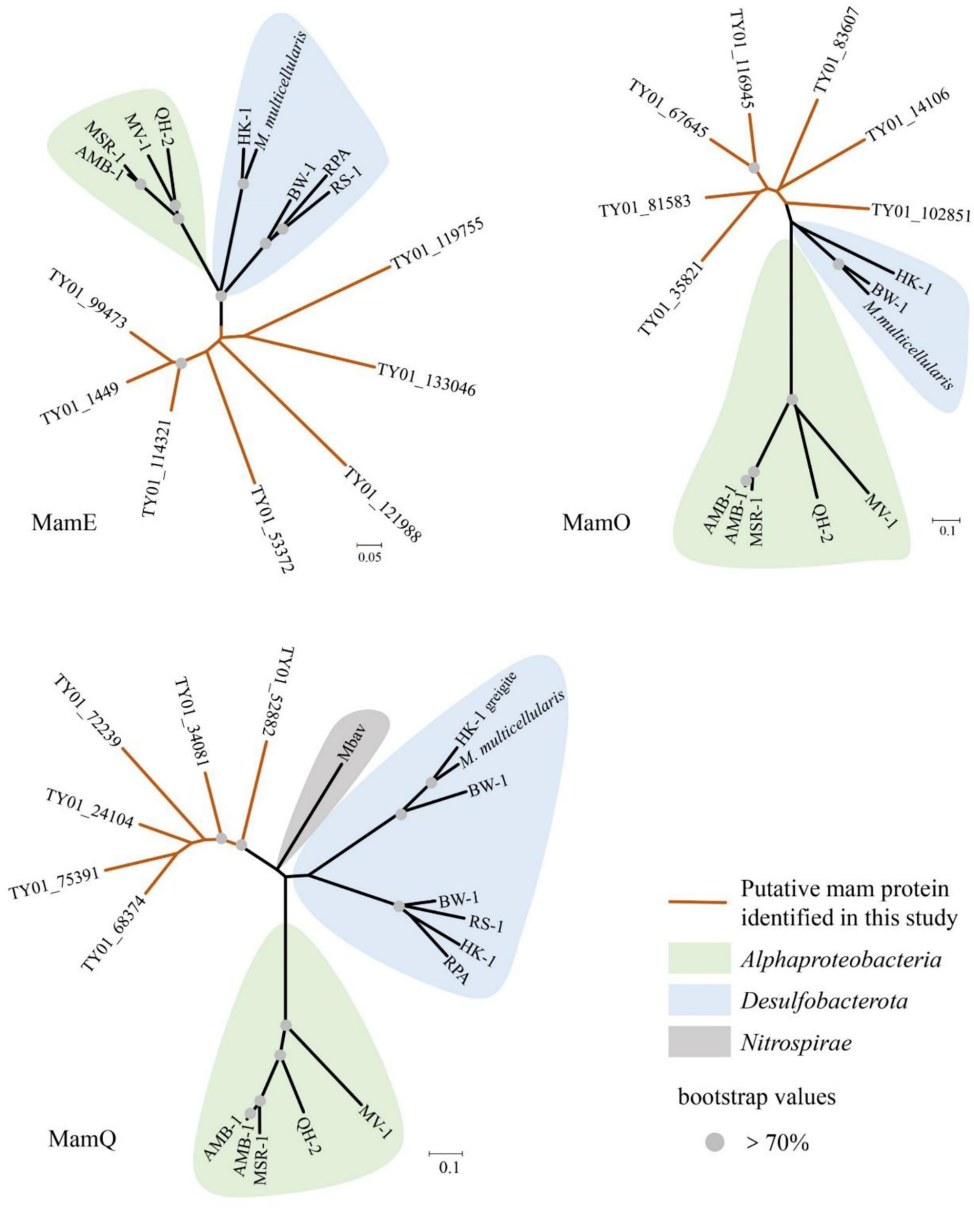
Neighbor-joining tree of homologous sequences of MamE, MamO, and MamQ protein from sediment of Tangyin hydrothermal field. HK-1, *Candidatus* Magnetomorum HK-1; RPA, *Candidatus* Magnetananas rongchenensis; BW-1, *Desulfamplus magnetovallimortis* BW-1; *M. multicellularis, Candidatus* Magnetoglobus multicellularis Araruama; RS-1, *Desulfovibrio magneticus* RS-1; QH-2, *Magnetospira* sp. QH-2; MV-1, *Magnetovibrio blakemorei* MV-1; AMB-1, *Magnetospirillum magneticum* AMB-1; MSR-1, *Magnetospirillum gryphiswaldense* MSR-1; Mbav, *Candidatus Magnetobacterium bavaricum*.

In our study, 16S rRNA gene analysis showed that MTB affiliated with *Desulfobacterota, Alphaproteobacteria*, and *Nitrospirae* had been found. FORC diagrams have no central ridge and the coercivity obtained from FORC is smaller than 20 mT, which is different from common FORCs of magnetofossils (Jovane et al., 2012). Vali and Kirschvink (1989) found partial magnetofossil dissolved in deep-sea sediment. Particle corrosion and clumping probably decreased the coercivity and increased the level of interparticle interaction, which is shown in our FORC diagram (Figure 3A). We also observed that partial magnetofossils experienced moderate corrosion in our sample (Supplementary Figure S1). Moreover, the diversity of magnetofossil was also affected by early diagenesis (Rodelli et al., 2019; Yamazaki, 2020). Yamazaki (2020) concluded that bullet-shaped magnetofossils were dissolved easier than prismatic ones, which may explain that the proportions of bullet-shaped magnetofossils are only 24.8% in our sample (Figure 3D). According to the diversity of magnetofossils observed from TEM images, the ancient MTB living in the Tangyin hydrothermal field of the Okinawa Trough might belong to *Alphaproteobacteria, Etaproteobacteria, Gammaproteobacteria, Desulfobacterota, Nitrospirae*, and the candidate phylum *Omnitrophica* (Figure 4) (Amor et al., 2020; Liu et al., 2021a,b). Moreover, we had detected some homologous sequences of magnetosome genes, and these homologous magnetosome genes did not form a complete magnetosome gene cluster. However, these homologous sequences have the same domain as the known magnetosome genes. This indicates they may have the same functions, and these homologous sequences could be magnetosome genes. Then, the phylogenetic trees of MamE, O, and Q protein sequences showed that these homologous sequences are evolutionarily distinct from known MTB (Figure 6). The homologous sequences of MamE and MamO representative sequences were homologous to *Desulfobacterota*. These branches were far from known MTB in the phylogenetic tree. Whereas, compared with the known MTB, most of these homologous Mam protein sequences have conserved regions (Supplementary Figure S2). Therefore, the MamE, O, and Q in hydrothermal have different evolutionary statuses and show high diversity. Of course, in consideration of these homologous sequences having big differences evolutionarily from known MTB, the homologous sequences of MamE and mamO are not necessarily *Desulfobacterota* either; they might be originated from other MTB identified by 16S rRNA gene sequences shown in Figure 2 (*Alphaproteobacteria* or *Nitrospirae)*. Meanwhile, the possibility that they are paralogous genes or pseudogenes from non-MTB cannot be ruled out. Nevertheless, combining the 16S rRNA gene results with the magnetofossils, we are more inclined that these are magnetosome genes from MTB. Here, our analysis of MTB-related 16S rRNA gene sequences, magnetofossils produced by MTB, and homologous sequences of magnetosome genes indicate that MTB could inhabit the deep-sea hydrothermal sediments.

Our analysis revealed that 65.3% of the 16S rRNA gene sequence reads associated with MTB belonged to OTU1477, which is affiliated with the MMP branch. All MMP-related 16S rRNA gene sequence reads accounted for 96.2% of all MTB-related reads. Moreover, the homologous sequences of magnetosome genes are most similar to known MTB belonging to *Desulfobacterota*, and MMPs accounted for 44.2% of all magnetosome gene homology sequences. The proportions of bullet-shaped magnetic particles are higher than those of MTB magnetosomes from the intertidal zone of Huiquan Bay in Qingdao (unpublished data) (24.8% vs. 4.0%). Bullet-shaped magnetosomes have only been found in the MTB belonging to *Desulfobacterota, Nitrospirae*, and the candidate phylum *Omnitrophica* (Kolinko et al., 2012; Chen et al., 2015; Li et al., 2015; Qian et al., 2019). Interestingly, both rock magnetic results and TEM analysis indicate that the abundance of BH components (bullet and elongated prisms) is ~1.7 times higher than that of BS components (cuboctahedron and short prisms) (Figures 3B,D). All the homologous sequences of *mam* genes identified herein correspond to the core genes of magnetosome synthesis. Given this, 16S rRNA gene sequences, magnetofossils, and homologous sequences of magnetosome genes consistently indicate that MMPs might be the dominant species among the MTB in this region. The hydrothermal field is an anoxic environment and is rich in reducing sulfides and sulfates (Orcutt et al., 2011). Previous research found that sulfate-reducing bacteria were the dominant species of the microbial community in the Tangyin hydrothermal field (Wang et al., 2018). Among the MTB, MMPs are anaerobic sulfate-reducing bacteria (Kolinko et al., 2014). Therefore, MMPs may represent the dominant group of MTBs at the sampled location, likely reflecting their adaptation to the hydrothermal environment.

As we all know, the hydrothermal zone is a candidate place for the origin of life (Trolard et al., 2022). Meanwhile, although the origins of MTB remain unclear, previous research has shown that the MTB of *Nitrospirae* and *Proteobacteria* differentiated near the Archean, and this suggests that MTB has existed at least in the Archean Eon (Lin et al., 2017). The geochemical conditions in the hydrothermal zone are thought to be similar to those on the early Earth (Baross and Hoffman, 1985; Trolard et al., 2022). The evolution of MTB here may be different from known MTB, which has been implied by the phylogenetic tree of 16S rRNA and homolog sequences of magnetosome genes. The dominant MTB in the hydrothermal field may represent a particular MTB species associated with the early Earth-like environment. There are microorganisms of ancient origin in hydrothermal vents (Takai and Nakamura, 2011), and MMPs are also an important model for studying evolution in prokaryotes (Keim et al., 2007). If MMPs are present in the hydrothermal field, the MMPs may be a potential model microorganism for understanding the early evolution of life on Earth.

## Conclusion

In this study, a total of 709 MTB-related 16S rRNA gene sequence reads were found in the Tangyin hydrothermal field. The 20 related OTUs represent *Desulfobacterota, Alphaproteobacteria*, and *Nitrospirae*. MMPs represented the largest number total of 121 homologous magnetosome gene sequences were annotated. The results collectively suggest that MTB exists in the Tangyin hydrothermal field and MMPs might be the dominant MTB in this region.

## Data Availability Statement

The datasets presented in this study can be found in online repositories. The names of the repository/repositories and accession number(s) can be found below: https://www.ncbi.nlm.nih.gov/genbank/, OM108105-OM108124 and https://www.ncbi.nlm.nih.gov/genbank/, PRJNA514953.

## Author Contributions

TX, WYZ, and WCZ designed the research. MY and X-HZ collected the sample and performed the metagenomic sequencing. WYZ and MY carried out a metagenomic analysis. SC carried out rock magnetic measurement and TEM experiments. SC and KH carried out the magnetism and TEM data analysis. SC, WYZ, KH, TX, and WCZ carried out data and statistical analysis. SC, TX, WYZ, WCZ, KH, and L-FW prepared the manuscript. All authors participated in the discussion of the results. All authors contributed to the article and approved the submitted version.

## Funding

This study was supported financially by the National Natural Science Foundation of China (U1706208 and 41976137).

## Conflict of Interest

The authors declare that the research was conducted in the absence of any commercial or financial relationships that could be construed as a potential conflict of interest.

## Publisher's Note

All claims expressed in this article are solely those of the authors and do not necessarily represent those of their affiliated organizations, or those of the publisher, the editors and the reviewers. Any product that may be evaluated in this article, or claim that may be made by its manufacturer, is not guaranteed or endorsed by the publisher.
