# Supplementary material for: Metagenomic and Microscopic Analysis of Magnetotactic Bacteria in Tangyin Hydrothermal Field of Okinawa Trough
Source: Front Microbiol. 2022 Jun 10;13:887136. doi: 10.3389/fmicb.2022.887136 (PMC9226615; doi:10.3389/fmicb.2022.887136)
Supplement: Supplementary file 3 [file Presentation_1.PPTX]

## Slide 1
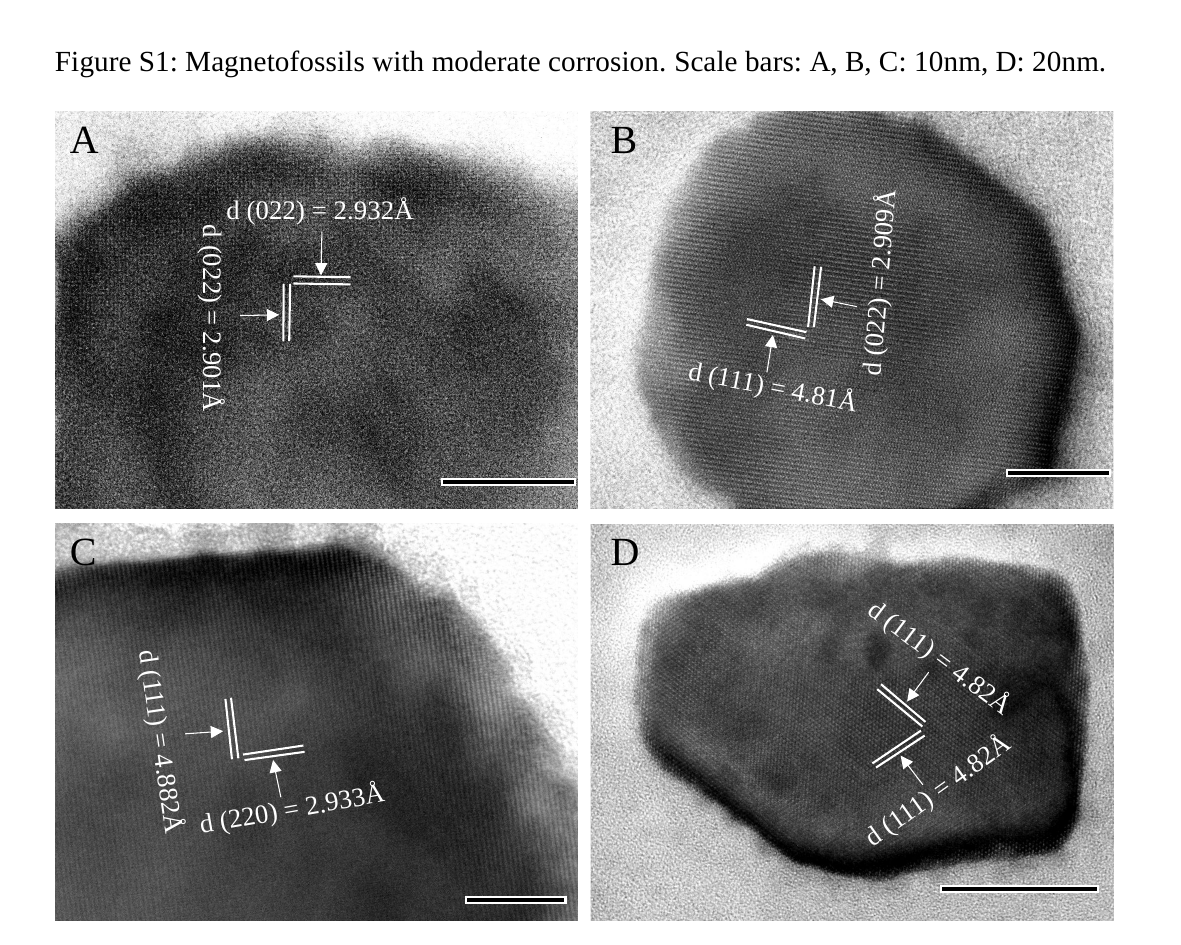

Figure S1: Magnetofossils with moderate corrosion. Scale bars: A, B, C: 10nm, D: 20nm.
A
B
d (022) = 2.932Å
d (022) = 2.901Å
d (022) = 2.909Å
d (111) = 4.81Å
C
D
d (111) = 4.882Å
d (220) = 2.933Å
d (111) = 4.82Å
d (111) = 4.82Å
