# Supplementary material for: Metagenomic and Microscopic Analysis of Magnetotactic Bacteria in Tangyin Hydrothermal Field of Okinawa Trough
Source: Front Microbiol. 2022 Jun 10;13:887136. doi: 10.3389/fmicb.2022.887136 (PMC9226615; doi:10.3389/fmicb.2022.887136)
Supplement: Supplementary file 4 [file Presentation_2.PPTX]

## Slide 1
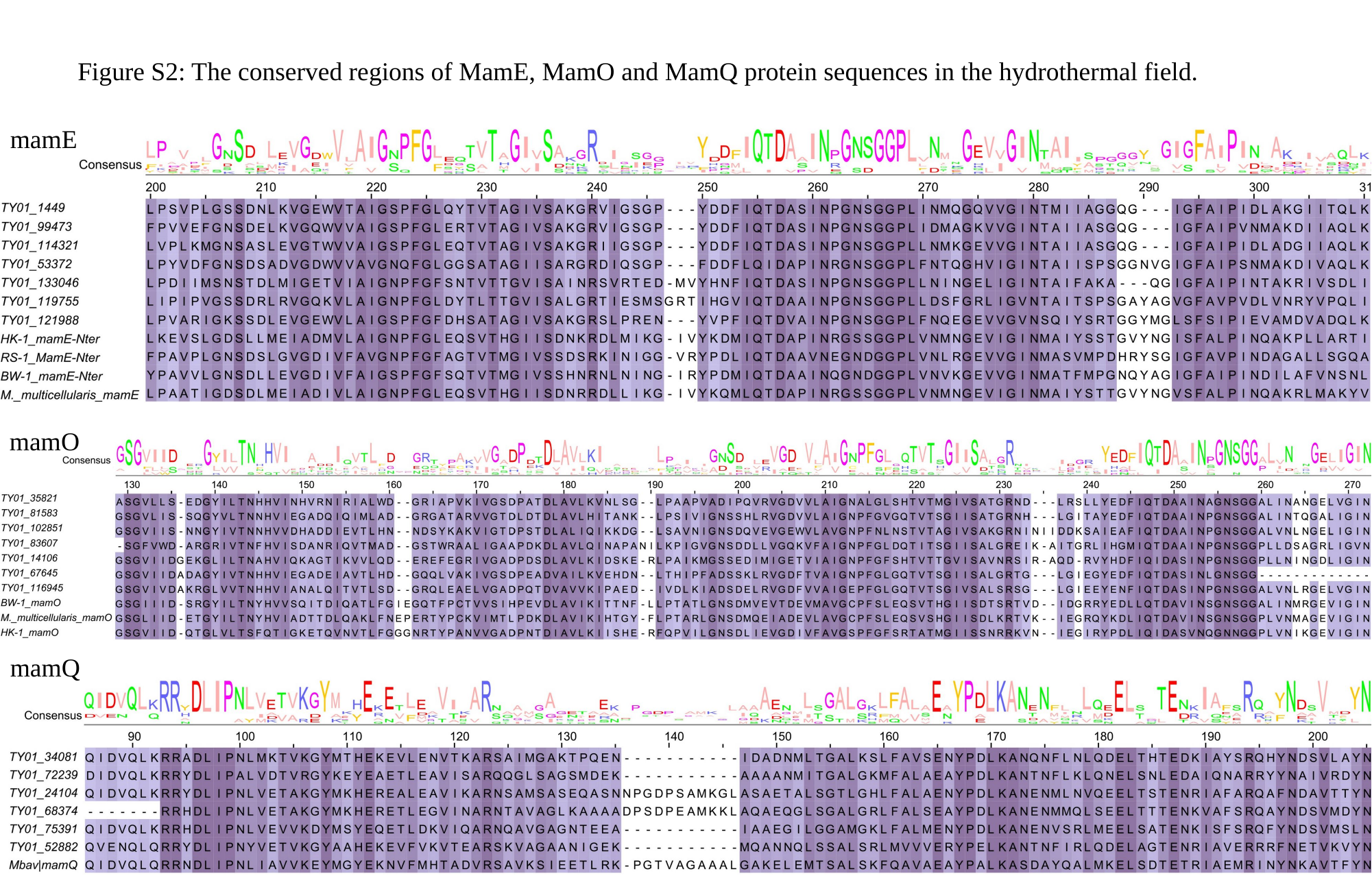

Figure S2: The conserved regions of MamE, MamO and MamQ protein sequences in the hydrothermal field.
mamE
mamO
mamQ
